# Experimental data of inorganic gel based smart window using silica sol–gel process

**DOI:** 10.1016/j.dib.2016.10.006

**Published:** 2016-10-22

**Authors:** Dayeon Jung, Woosuk Choi, Jun-Young Park, Ki Buem Kim, Naesung Lee, Yongho Seo, Hyun Sub Kim, Nak Kyoung Kong

**Affiliations:** a Faculty of Nanotechnology & Advanced Materials, HMC, and GRI, Sejong University, Seoul 143-747, South Korea; b R&D division, 150, Hyundaiyeonguso-ro, Namyang-eup, Hwaseong-si, Gyeonggi-do 445-706, South Korea

**Keywords:** Inorganic gel, Smart window, Silica sol–gel process, GDLC

## Abstract

In this article experimental data are presented for inorganic gel based smart window fabricated using silica sol–gel process. Parallel beam transmittances were measured as functions of voltages for samples fabricated with different concentrations of nitric acid. Spectroscopic transmittance data at different driving voltages for samples fabricated with different LC concentrations are shown. Transmittance spectra of the Si–Ti based gel-based-liquid-crystal (GDLC) device measured as different driving voltages were compared with those of PDLC. GDLC showed much lower operating voltages, 10–15 V, for on-state. Formation of the LC droplet in gelation process is illustrated. The methyl organic group surrounds LC droplets. Demonstration of GDLC based smart window showed the successful operation with low driving voltages. GDLC window shows clear color, even at off-state, compared with PDLC.

**Specifications Table**

**Table:** 

Subject area	*Physics*
More specific subject area	*Opto-electrics*
Type of data	*Images*
How data was acquired	Transmittance spectra using UV/IR spectrometer, *Photocurrent versus voltage measurement using photodiode, Microscope,*
Data format	*Filtered and analyzed*
Experimental factors	*Gel based liquid crystal sample as prepared*
Experimental features	*Transmittance, spectroscopy*
Data source location	*Seoul, South Korea*
Data accessibility	*Data is with the article*

**Value of the data**

• Parallel beam transmittances for GDLC can be used to optimize the concentration of nitric acid in fabrication process of GDLC.
• Spectroscopic transmittances for GDLC provide an experimental data to optimize the concentration of liquid crystal.
• Demonstration of GDLC mirror is to show application of transportation requiring its fast switching response.

## 1 Data

**Fig. 1:**
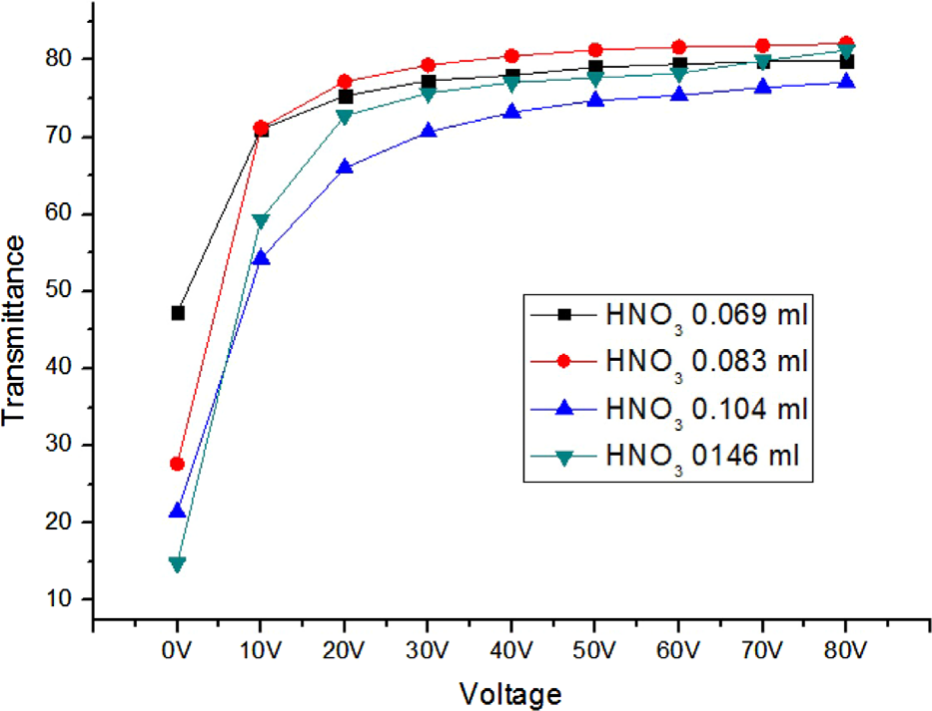
Parallel beam transmittances were measured as functions of voltage for samples fabricated with different concentrations of nitric acid. The sample with 0.146 ml shows the highest on/off contrast.

**Fig. 2:**
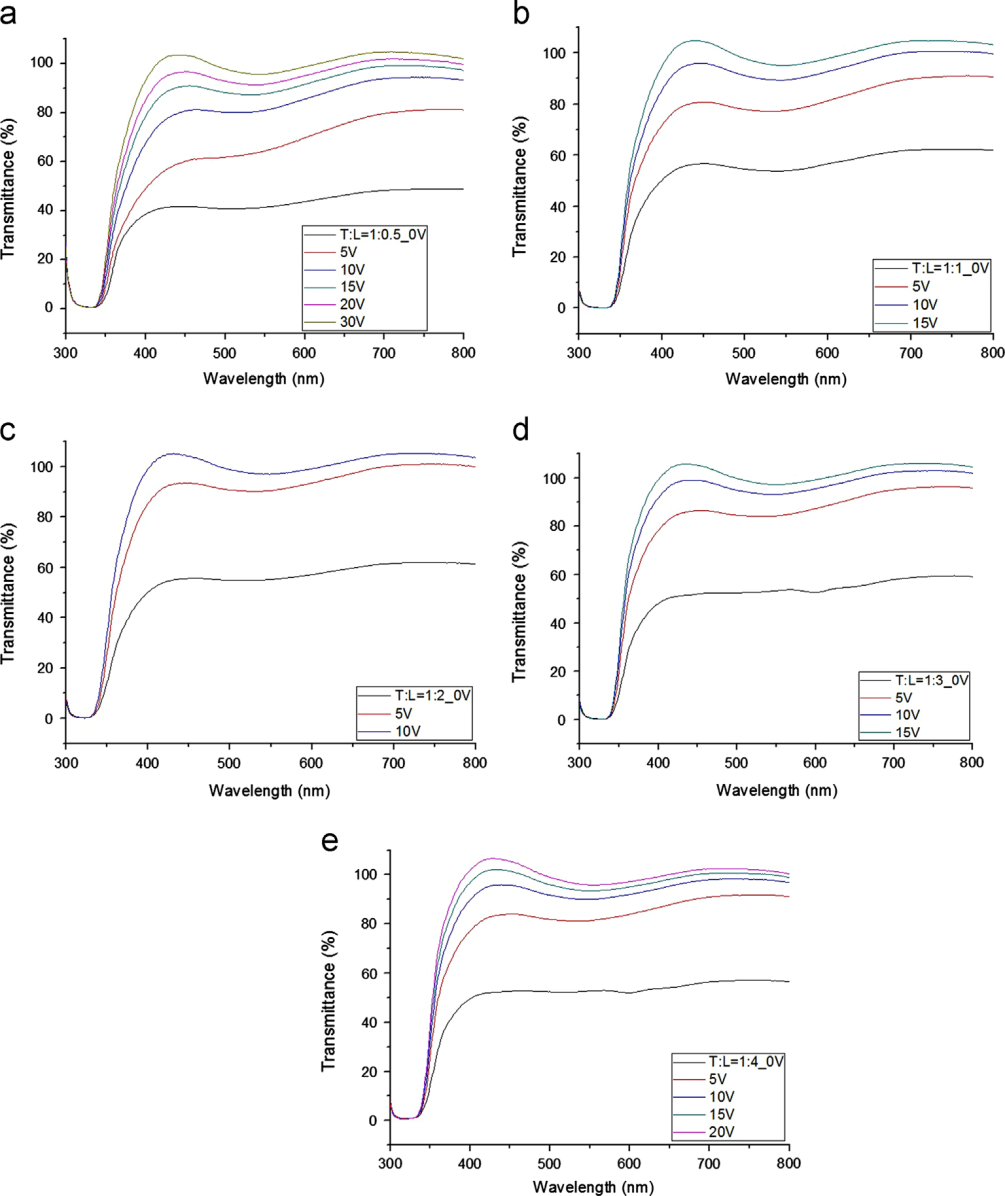
Spectroscopic transmittance data at different driving voltages for samples fabricated with different LC concentrations are shown. The concentration ratio between the mixed liquid and LC were (a) 1:0.5, (b) 1:1, (c) 1:2, (d) 1:3, and (e) 1:4.

**Fig. 3:**
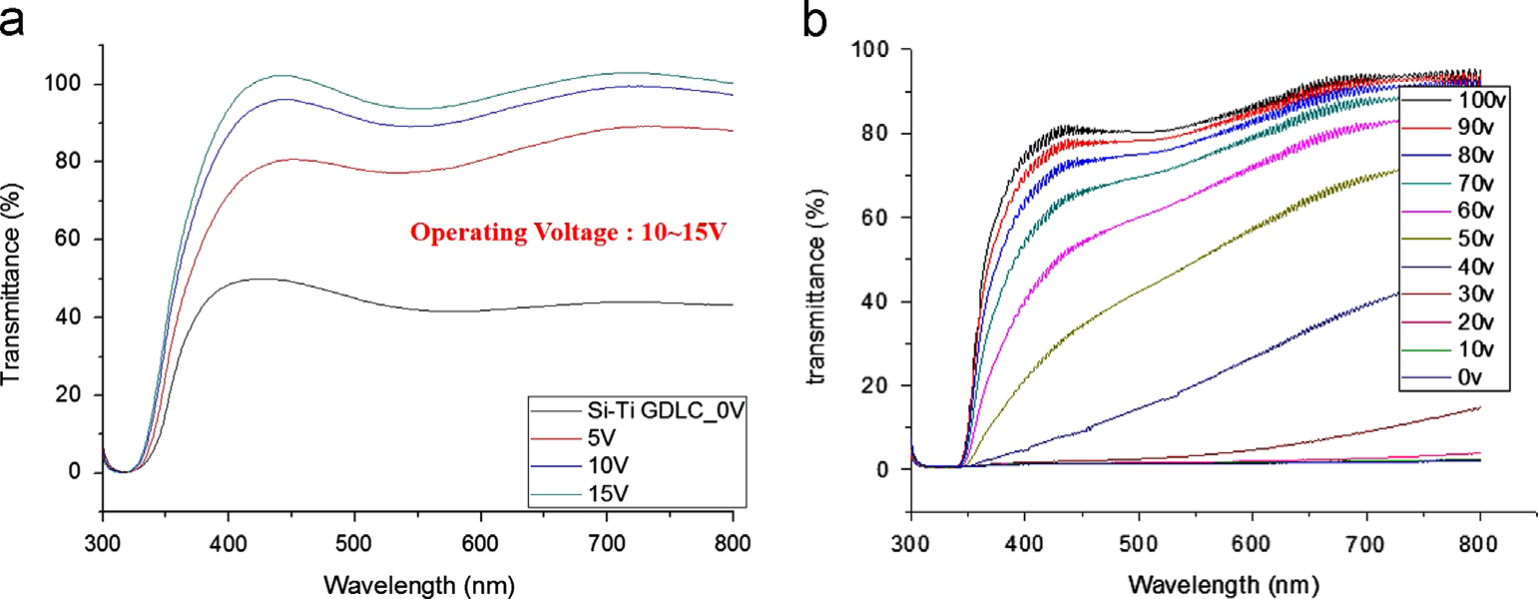
Transmittance spectra of the Si–Ti based GDLC device measured as different driving voltages were compared with those of PDLC. (a) GDLC showed much lower operating voltages, 10–15 V, for on-state. While (b) PDLC shows tilted slopes for intermediate voltages, GDLC displays flat spectra.

Transmittances for parallel beam with a laser were measured at different voltages for gel-based-liquid-crystal (GDLC) samples fabricated with different concentrations of nitric acid (Fig. 1). Spectroscopic transmittance data at different driving voltages for GDLCs fabricated with different LC concentrations are presented (Fig. 2). Transmittance spectra of the Si–Ti based GDLC device measured as different driving voltages were compared with those of PDLC [1,2]. GDLC showed much lower operating voltages, 10–15 V, for on-state. While PDLC shows tilted slopes for intermediate voltages, GDLC displays flat spectra (Fig. 3. SEM images of top and fractured surfaces were taken to inspect the microstructures of gel of GDLC (Fig. 7, Fig. 8).

## 2 Experimental design, materials and methods

**Fig. 4:**
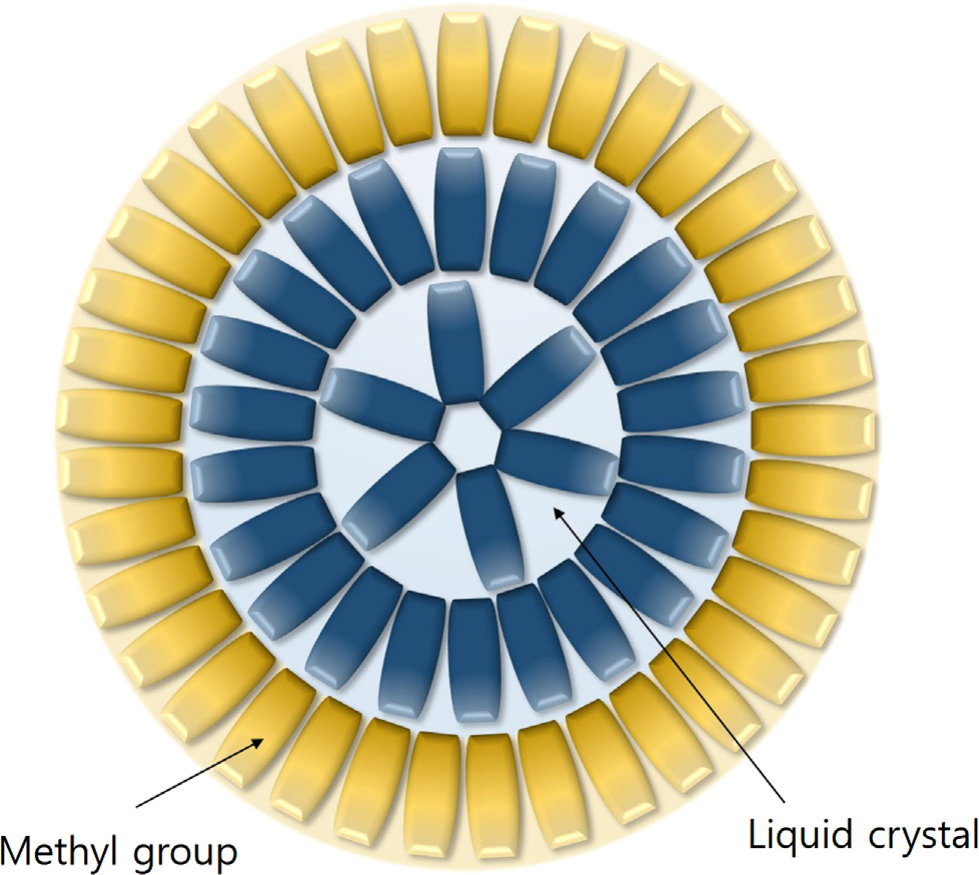
Formation of the LC droplet in gelation process is illustrated. The methyl organic group surrounds a LC droplet.

**Fig. 5:**
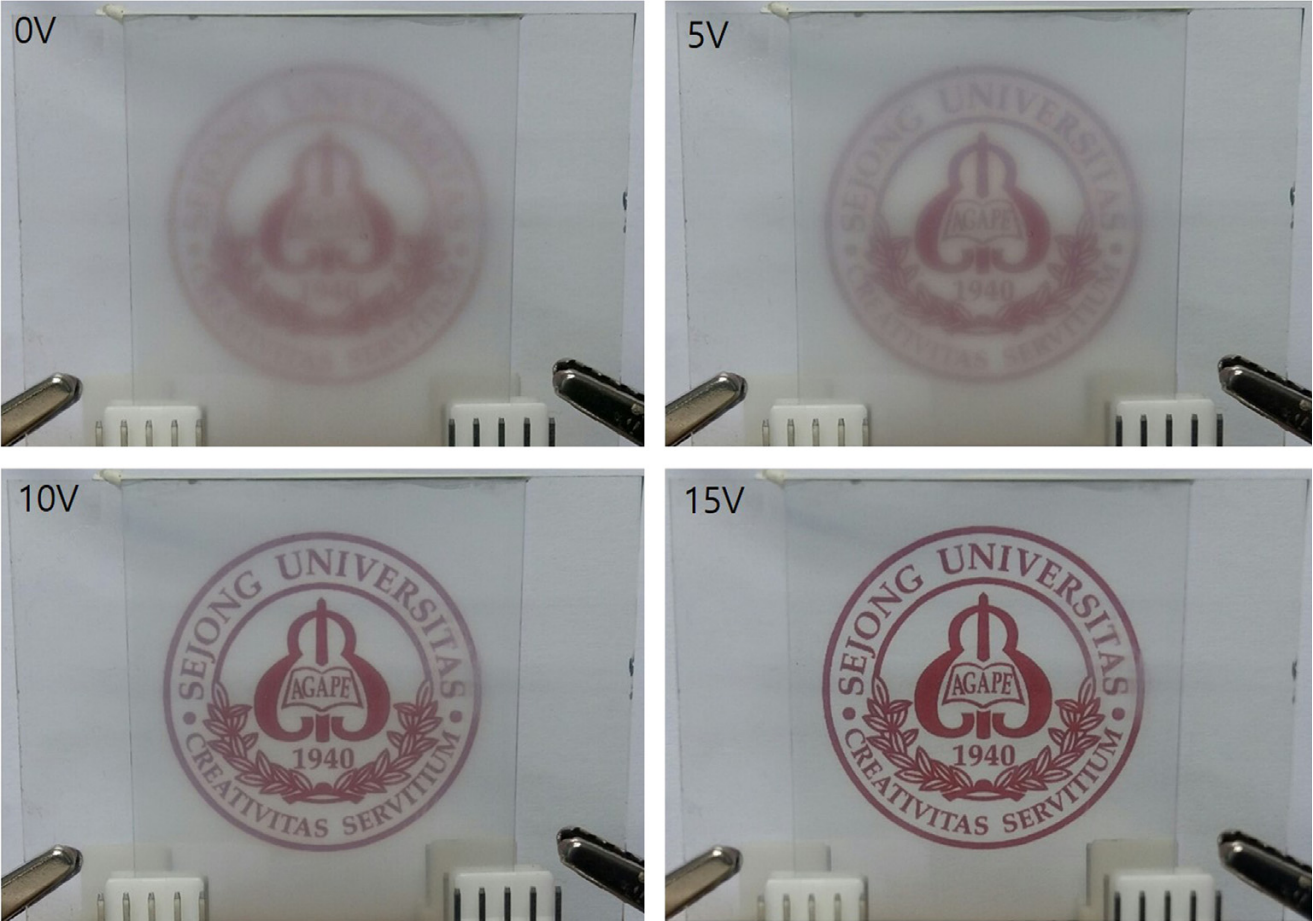
Demonstration of GDLC based smart window shows the successful operation with low driving voltages. GDLC window shows clear color, even at off-state.

**Fig. 6:**
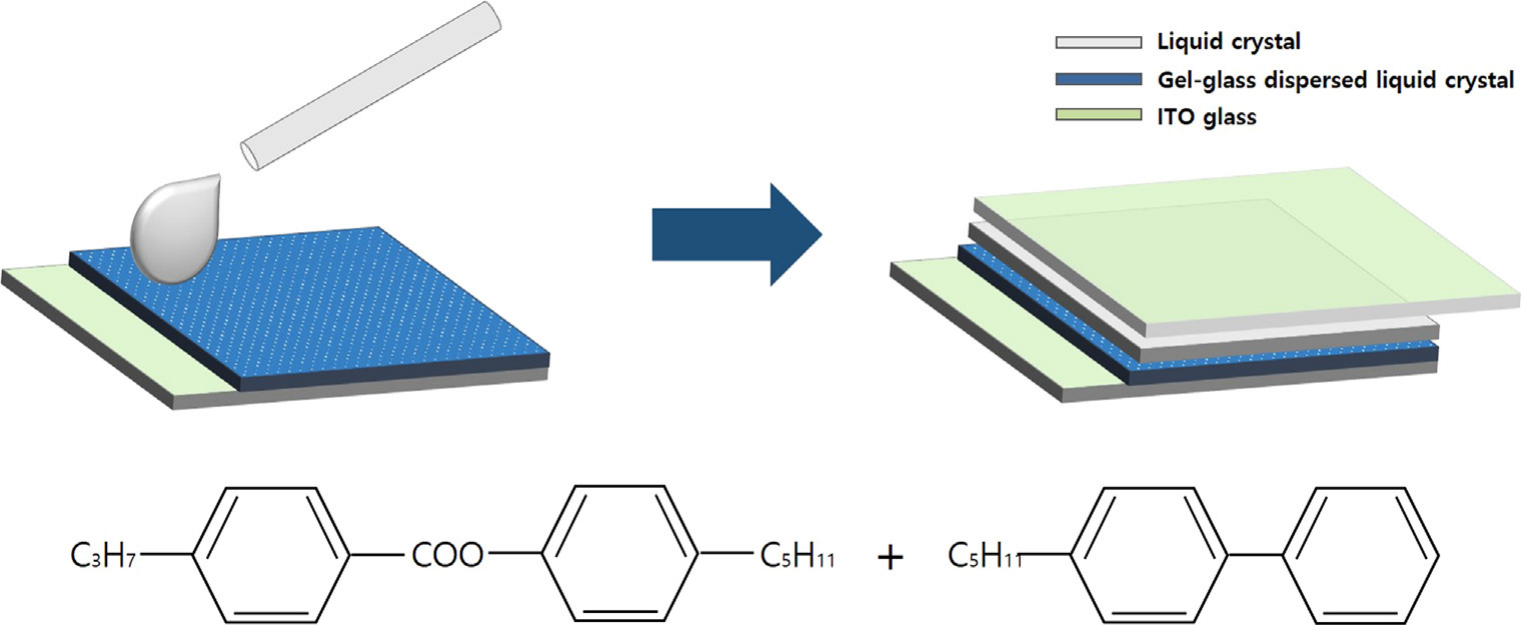
Schematic shows GDLC sandwich structure with additional LC coating. Chemical formula of LC used in this experiment is shown.

**Fig. 7:**
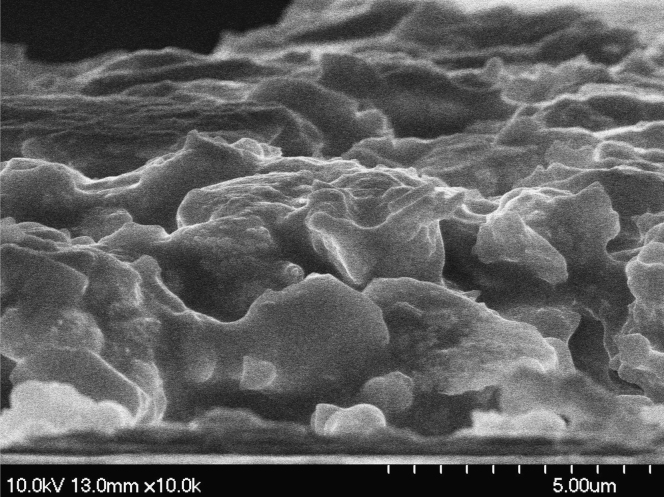
SEM image of fractured surface of GDLC.

**Fig. 8:**
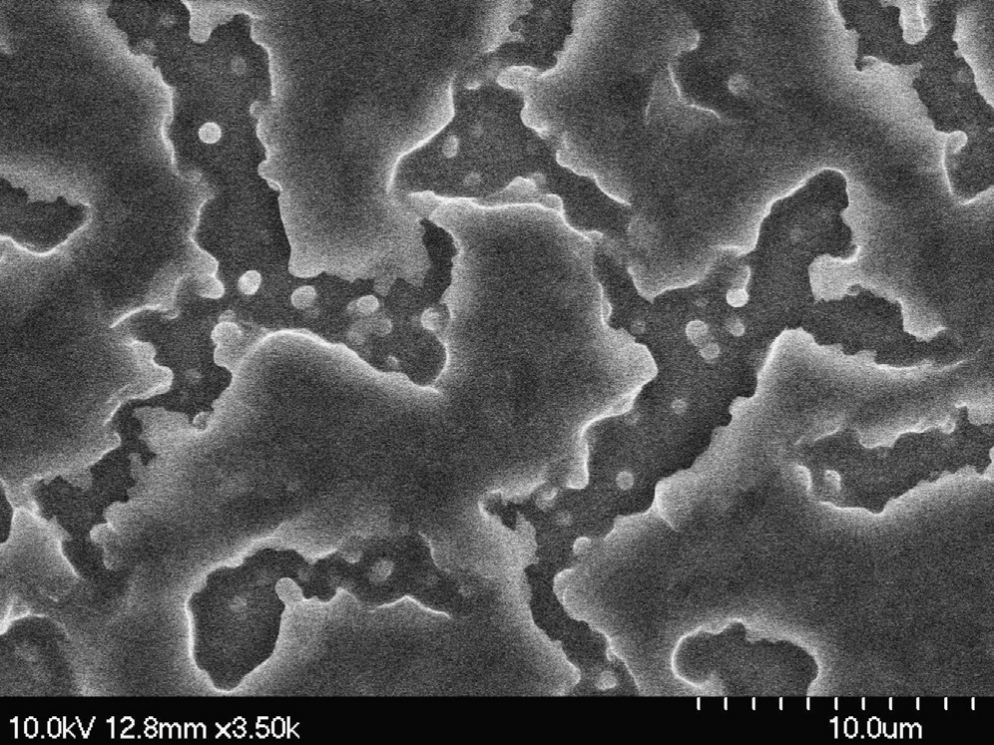
SEM image of top surface of GDLC.

Transmittance and reflectance of GDLC as functions of wavelength were measured with UV/vis spectroscopy (Cary 5000, Varian). Spectroscopic transmittance data were measured in the range of 300–800 nm wavelength. The parallel transmittance and response time were measured by a home-built setup composed of a photo diode and a laser diode (*λ*=635 nm). An optical microscope was used to investigate LC droplets size and distribution (Fig. 4, Fig. 5, Fig. 6).
