# Supplementary material for: Experimental data of inorganic gel based smart window using silica sol–gel process
Source: Data Brief. 2016 Oct 22;9:716–22. doi: 10.1016/j.dib.2016.10.006 (PMC5094103; doi:10.1016/j.dib.2016.10.006)
Supplement: Supplementary file 2 — Supplementary material [file mmc2.pdf]

Table S1 List of all substances with their chemical formula and role in the silica sol-gel process.

compound	name	chemical formula	role
Metal oxide	Tetra ethyl ortho-silicate (TEOS)	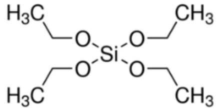	Inorganic gel matrix
	Triethoxymethylsilane (Me-TES)	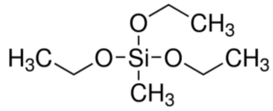	
	Titanium isopropoxide	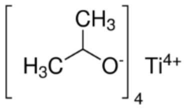	
water	Distilled ionized (DI)	H_2O	hydrolysis
solvent	Isopropyl alcohol (IPA)	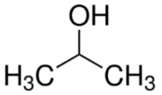	homogeneous solution
acid	Nitric acid (HNO_3)	HNO_3	catalysis reaction
additives	Acetylacetone	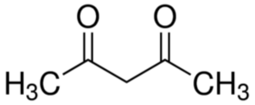	homogeneous reaction

2. Experimental Design, Materials and Methods

Transmittance and reflectance of GDLC as functions of wavelength were measured with UV/Vis spectroscopy (Cary 5000, Varian). Spectroscopic transmittance data were measured in the range of 300 to 800 nm wavelength. The parallel transmittance and response time were measured by a home-built setup composed of a photo diode and a laser diode (λ = 635 nm). An optical microscope was used to investigate LC droplets size and distribution.

Acknowledgements

This work was supported by the Human Resources Development of the Korea Institute of Energy Technology Evaluation and Planning (KETEP) grant funded by the Korean government's Ministry of Trade, Industry & Energy (No. 20154030200630). Also, this work is supported by the industrial research innovation program (10051701), funded by the Ministry of Trade,
